# Development of a duodenal gallstone ileus with gastric outlet obstruction (Bouveret syndrome) four months after successful treatment of symptomatic gallstone disease with cholecystitis and cholangitis: a case report

**DOI:** 10.1186/1752-1947-4-376

**Published:** 2010-11-23

**Authors:** Arnd Giese, Jürgen Zieren, Guido Winnekendonk, Bernhard F Henning

**Affiliations:** 1 Department of Internal Medicine, Gastroenterology Unit, Marienhospital, Ruhr-University Bochum, Hölkeskampring 40, 44625 Herne, Germany; 2 Department of Surgery, Marienhospital, Ruhr-University Bochum, Hölkeskampring 40, 44625 Herne, Germany; 3 Department of Radiology, Marienhospital, Ruhr-University Bochum, Hölkeskampring 40, 44625 Herne, Germany

## Abstract

**Introduction:**

Cases of gallstone ileus account for 1% to 4% of all instances of mechanical bowel obstruction. The majority of obstructing gallstones are located in the terminal ileum. Less than 10% of impacted gallstones are located in the duodenum. A gastric outlet obstruction secondary to a gallstone ileus is known as Bouveret syndrome. Gallstones usually enter the bowel through a biliary enteral fistula. Little is known about the formation of such fistulae in the course of gallstone disease.

**Case presentation:**

We report the case of a 72-year-old Caucasian woman born in Germany with a gastric outlet obstruction due to a gallstone ileus (Bouveret syndrome), with a large gallstone impacted in the third part of the duodenum. Diagnostic investigations of our patient included plain abdominal films, gastroscopy and abdominal computed tomography, which showed a biliary enteric fistula between the gallbladder and the duodenal bulb. Our patient was successfully treated by laparotomy, duodenotomy, extraction of the stone, cholecystectomy, and resection of the fistula in a one-stage surgical approach. Histopathological examination showed chronic and acute cholecystitis, with perforated ulceration of the duodenal wall and acute purulent inflammation of the surrounding fatty tissue. Four months prior to developing a gallstone ileus our patient had been hospitalized for cholecystitis, a large gallstone in the gallbladder, cholangitis and a small obstructing gallstone in the common biliary duct. She had been treated with endoscopic retrograde cholangiopancreatography, endoscopic biliary sphincterotomy, balloon extraction of the common biliary duct gallstone, and intravenous antibiotics. At the time of her first presentation, abdominal ultrasound and endoscopic examination (including esophagogastroduodenoscopy and endoscopic retrograde cholangiopancreatography) had not shown any evidence of a biliary enteral fistula. In the four months preceding the gallstone ileus our patient had been asymptomatic.

**Conclusion:**

In patients known to have gallstone disease presenting with symptoms of ileus, the differential diagnosis of a gallstone ileus should be considered even in the absence of preceding symptoms related to the gallbladder disease. Gallstones large enough to cause intestinal obstruction usually enter the bowel by a biliary enteral fistula. During the formation of such a fistula, patients can be asymptomatic.

## Introduction

Gallstone ileus accounts for approximately 1% to 4% of all cases of mechanical bowel obstruction. However, in the population over the age of 65 it is the cause of 25% of non-strangulated small bowel obstructions. Diagnosis is often delayed and mortality is high, ranging at 15% to 18%, which may also reflect the age and comorbidity of affected patients [1]. Gallstones usually enter the bowel through a biliary enteric fistula, which complicates 2% to 3% of cases of cholecystolithiasis with associated episodes of cholecystitis [2]. Due to the sedimentation of intestinal content, gallstones increase in diameter as they pass the bowel. The majority of obstructing gallstones are located in the terminal ileum (50% to 75%), followed by the proximal ileum and jejunum (20% to 40%). Gallstones impacted in the duodenum account for less than 10% [3]. A gastric outlet obstruction secondary to an impacted gallstone in the duodenum or pylorus is called Bouveret syndrome. It was first described in 1896 by the French internist Leon Bouveret, and up to 1999 only 175 cases had been described in the medical literature [4]. Our case is a rare description of Bouveret syndrome developing four months after successful treatment of symptomatic gallstone disease and after a four-month period with no symptoms.

## Case report

A 72-year-old Caucasian woman born in Germany was admitted to our hospital with acute onset of nausea, vomiting and diffuse abdominal pain. Her only medications were metoprolol tartate and ramipril for arterial hypertension and chronic compensated heart failure. Physical examination was normal apart from diffuse pain on abdominal palpation. There were no signs of peritonitis. Laboratory findings (Table 1) included a white blood count of 14.3 cells/nL, an elevated C-reactive protein (CRP) level of 25.9 mg/dL, mildly elevated plasma aspartate aminotransferase and alanine aminotransferase (AST and ALT) levels of 51 U/L and 83 U/L, a moderate elevation of the γ glutamyl transpeptidase (GGT) level of 487 U/L and an alkaline phosphatase (AP) level of 368 U/L. Her total bilirubin level was elevated to 1.17 mg/dL and her serum creatinine level was 1.84 mg/dL. An abdominal ultrasonography scan showed thickening and edema of the gallbladder (GB) wall (12 mm), double wall sign, the presence of a large gallstone and a local hypoechogenic mass in the GB adhering to the GB wall with no signs of vascularization on color flow imaging. The common biliary duct (CBD) was dilated to 10 mm. Endoscopic retrograde cholangiopancreatography (ERCP) performed on the day of admission revealed a normal pancreatic duct and a small pigmented gallstone of the CBD that was extracted with an extraction balloon after endoscopic biliary sphincterotomy. Esophagogastroduodenoscopy (EGD) findings were normal without any signs of perforation or fistula. Under antibiotic treatment (ceftriaxon 2 g intravenously a day and metronidazole 400 mg intravenously four times a day for 10 days), our patient recovered completely. Her white blood count normalized and CRP and GGT levels fell (CRP 1.6 mg/dL, GGT 284 U/L two days before discharge). She was discharged after 11 days. After discharge our patient continued her antibiotic treatment (cefuroxim 500 mg orally twice a day and metronidazole 500 mg orally three times a day) for another four days.

**Table 1:** Laboratory data for blood at admission

	Normal range	First admission	Second admission
WBC	4.0 to 10.0 cells/nL	14.3 cells/nL	11.7 cells/nL

Segmented cells		85%	NA

Lymphocytes	25% to 40%	6%	NA

Monocytes	2% to 6%	8%	NA

Eosinophils	2% to 7%	0%	NA

Basophils	0% to 1%	1%	NA

ESR after 1 hour	6 to 11 mm	104 mm	44 mm

RBC	4.1 to 5.1 cells/pL	4.53 cells/pL	5.16 cells/pL

Hemoglobin	12 to 16 g/dL	13.5 g/dL	14.2 g/dL

Hematocrit	35% to 45%	39.7%	42.2%

Platelets	140 to 440 cells/nL	303 cells/nL	360 cells/nL

Bilirubin (total)	< 1.2 mg/dL	1.68 mg/dL	0.97 mg/dL

Bilirubin (conjugated)	< 0.5 mg/dL	1.17 mg/dL	NA

Creatinine	0.5 to 0.9 mg/dL	1.84 mg/dL	1.12 mg/dL

AP	40 to 150 U/L	368 U/L	97 U/L

GGT	9 to 39 U/L	487 U/L	84 U/L

AST	5 to 31 U/L	51 U/L	29 U/L

ALT	0 to 34 U/L	83 U/L	12 U/L

LDH	< 243	208 U/L	252 U/L

Potassium	3.5 to 5.1 mmol/L	3.60 mmol/L	3.93 mmol/L

Sodium	136 to 145 mmol/L	137 mmol/L	144 mmol/L

Lipase	8 to 78 U/L	32 U/L	43 U/L

CRP	< 0.5 mg/dL	25.93 mg/dL	0.83 mg/dL

INR	0.85 to 1.17	0.87	0.99

pTT	25 to 40 seconds	37 seconds	32 seconds

ALT = alanine aminotransferase; AP = alkaline phosphatase; AST = aspartate aminotransferase; CRP = C-reactive protein; ESR = erythrocyte sedimentation rate; GGT = γ glutamyl transpeptidase; INR = international normalized ratio; LDH = lactate dehydrogenase; NA = not applicable; pTT = partial thromboplastin time; RBC = red blood cell count; WBC = white blood cell count.

As she remained asymptomatic, our patient did not attend the cholecystectomy scheduled two months after hospital discharge. Instead, four months after her initial discharge, she re-presented to our hospital with abdominal right upper quadrant (RUQ) pain and repeated post-prandial vomiting.

Physical examination at this time showed RUQ pain with no local tenderness or other signs of peritonitis. At admission her blood pressure was 140/90 mmHg and her body temperature was 37°C. The laboratory findings at the time of her second admission revealed a white blood count of 11.7 cells/nL, a slightly elevated CRP level of 0.83 mg/dL and normal liver test results apart from an elevated GGT level of 84 U/L.

**Figure 1:**
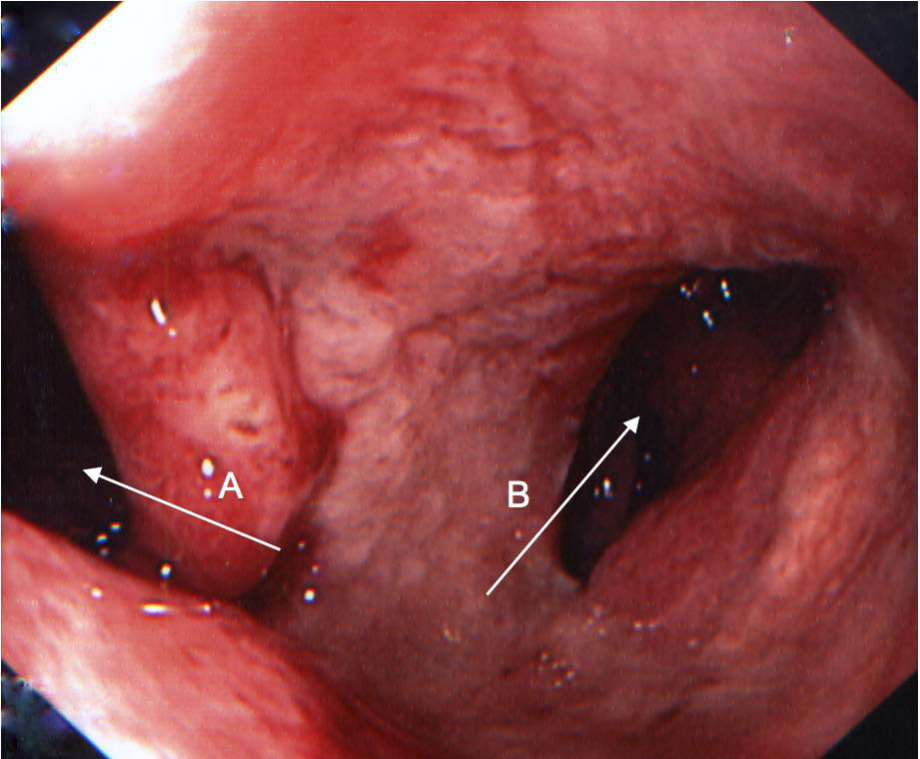
**Endoscopic view of the duodenal bulb**. Arrow A: view into the descending duodenum. Arrow B: biliary enteral fistula.

EGD was performed, during which 1.5 L of gastric content was removed by endoscopic suction. A fistula leading into a cavity of 2 cm diameter was detected just distal of the pyloric sphincter on the dorsal wall of the duodenal bulb, as well as some small fibrin-covered erosions on the anterior wall of the duodenal bulb (Figure 1).

**Figure 2:**
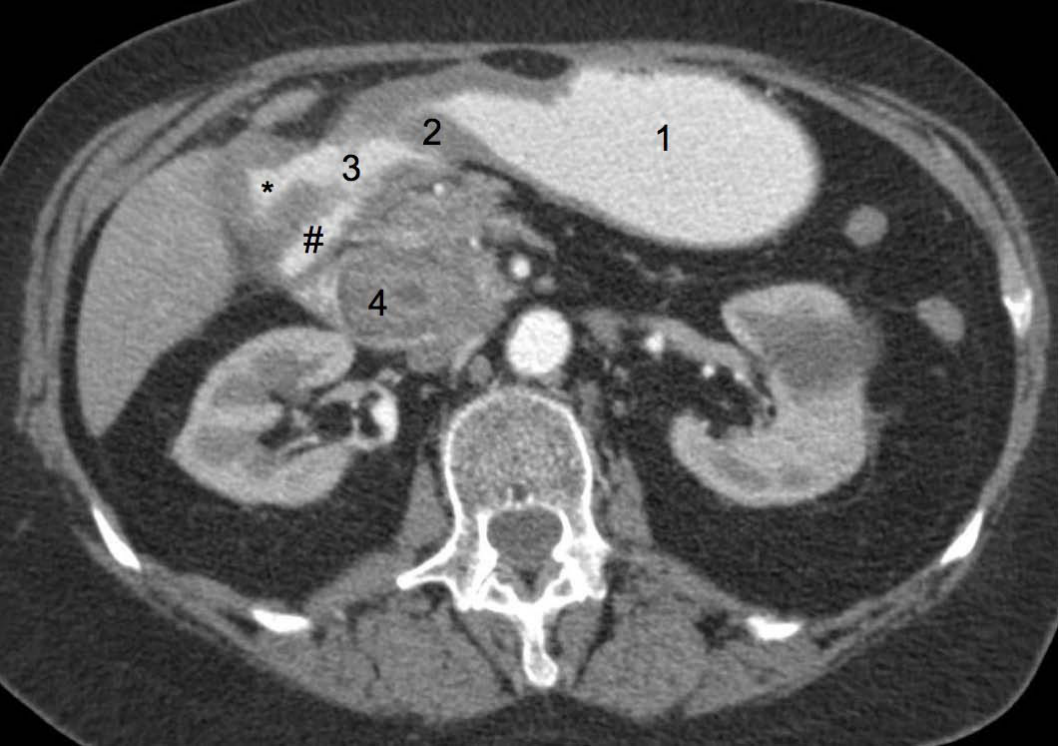
**Abdominal computed tomography (CT) scan with intravenous and oral contrast enhancement**. Labels on the figure are as follows. 1: Contrast material in the lumen of the stomach. 2: Pyloric sphincter. 3: Contrast material in the lumen of the duodenal bulb. 4: Gallstone impacted in the lumen of the third part of the duodenum. *: Contrast material in the lumen of the gallbladder (notice thickening of the wall of the gallbladder and communication with the duodenal bulb). #: Contrast material in the lumen of the descending duodenum.

Chest radiography results revealed an absence of pulmonary infiltrate. On plain abdominal film no signs of ileus, pneumobilia or free air could be detected. A CT scan of the abdomen with oral and intravenous contrast (Figure 2) revealed a gallstone ileus with a 4 cm × 3 cm gallstone in the third part of the duodenum associated with a fistula between the GB and the duodenal bulb, as well as minimal pneumobilia. The impacted gallstone was surgically removed by laparotomy and duodenotomy. It measured 5 cm × 3 cm. Cholecystectomy and excision of the fistula was performed. A histopathologic examination revealed a gallbladder with chronic and acute cholecystitis, high-grade chronic granulating xanthomatous and purulent pericholecystitis with a foreign body granuloma. The duodenal wall excision showed high-grade chronic fibrosing and acute ulcerating inflammation with perforated ulceration as well as chronic and acute purulent inflammation of the surrounding fatty tissue. Postoperative duodenal leakage or persistence of duodenal obstruction was ruled out by a contrast swallow. Our patient's recovery was uneventful. At seven weeks after discharge (eight weeks after surgery) she was doing well, and was able to continue her usual daily activities immediately after discharge.

## Discussion

This case report is the first published observation of this particular course of gallstone disease. In our patient, a duodenal gallstone ileus developed four months after a cholecystitis associated with a large gallstone in the GB, a small obstructing gallstone in the CBD, and cholangitis. It is striking that the formation of the biliary enteral fistula must have taken place in an asymptomatic period of four months. Fistula formation and dislocation of a gallstone from the GB into the duodenum happened even after sufficient biliary drainage and antibiotic treatment during our patient's first hospitalization.

It is generally believed that pericholecystic inflammation after cholecystitis, as well as pressure necrosis by the gallstone against the biliary wall, may lead to formation of a biliary enteric fistula. Fistula formation is a complication of 2% to 3% of all cases of cholelithiasis with associated episodes of cholecystitis [2]. Obstruction of the biliary systems is known to promote cholecystitis. It also seems to play a role in the formation of a biliary enteric fistula. In a large series reported by Beltran *et al.*, 89.5% of patients with cholecystoenteric fistulae were also found to have a CBD obstruction caused by an extrinsic compression from an impacted stone in the cystic duct, known as Mirizzi syndrome [5]. A biliary enteric fistula provides a passage for large gallstones to enter the bowel and eventually cause gallstone ileus. Biliary enteric fistulae are comprised of 60% cholecystoduodenal fistulae, but cholecystocolonic and cholecystogastric fistulae can also lead to a gallstone ileus [6]. Although a gallstone ileus is usually preceded by the formation of a biliary enteric fistula, there also exists a description in the literature of a gallstone ileus after endoscopic biliary sphincterotomy [7] with a large extracted stone causing gallstone ileus. We do not believe that this was the pathomechanism in our patient since the migration of the large stone through the fistula between the GB and the duodenum seems to be more likely than a passage through the CBD.

ERCP performed during our patient's first hospitalization revealed only a small gallstone in the CBD. The histopathologic findings from our patient also support the theory of pericholecystitis leading to fistula formation. The hypoechogenic mass in the GB found at abdominal ultrasonography during our patient's first visit may have been a sign of granuloma formation. It could also correspond to GB sludge. Although early cholecystectomy seems to yield equivalent outcomes as delayed cholecystectomy [8], we decided to opt for a delayed cholecystectomy as our patient presented with cholangitis, severe inflammation, signs of serious local inflammation and elevated creatinine at the time of her first visit. As the situation corresponded to a moderate to severe (grade I to II) acute cholecystitis according to the Tokyo guidelines, this approach seems reasonable [9].

In our patient, plain abdominal films did not show pneumobilia or a gallstone. The diagnosis was made on the basis of the results of an abdominal CT scan and gastroscopy. However, a biliary enteral fistula and a gallstone ileus may also be seen by ultrasound imaging [10]. The therapeutic approach to our patient having gallstone ileus remains a subject of debate, mostly due to a lack of large prospective studies. Our patient recovered well after a laparotomy with simultaneous extraction of the gallstone, cholecystectomy and resection of the fistula. However, in the recent literature a high perioperative mortality rate of up to 35% is described. The high mortality is mainly attributed to the delay of time between first symptoms and admission, with an average of three to five days.

Possible strategies are a one-stage approach with enterotomy, cholecystectomy and resection of the fistula at once, or a two-stage approach with an emergency enterotomy to remove the obstructing gallstone and cholecystectomy after a period of recuperation. It seems reasonable to restrict the one-stage approach to clinically stable patients and to choose a two-stage approach in patients with severe cholecystitis and a high perioperative risk as a result of concomitant comorbidities [11]. For Bouveret syndrome, endoscopic extraction of the gallstone [12] has been described, as well as extracorporeal shockwave lithotripsy and argon plasma coagulation [13] or duodenotomy. As with more distal gallstone ileus the primary therapeutic goal should be to relieve the gallstone obstruction. In principle, laparoscopic treatment of gallstone ileus is possible and was initially considered for our patient. However, the location of gallstones along the entire length of the bowel, especially in the presence of obstruction, and a probably longer operation time may be problematic [14]. Also, laparoscopic extraction of large gallstones may cause problems. With the gallstone of our patient measuring 3 cm × 4 cm on a CT scan and because we were planning a one-stage surgery, we performed a laparotomy instead of choosing a laparoscopic approach.

## Conclusion

In a patient with gallstone disease with abdominal pain, nausea and vomiting, the possibility of a gallstone ileus leading to gastric outlet obstruction (Bouveret syndrome) should be considered. A CT scan of the abdomen can be helpful in making the diagnosis. Gastroscopy should be performed and may in some cases offer non-invasive treatment options. If the patient is not heavily compromised by the gallstone ileus itself or by comorbidities, a one-stage surgical approach with simultaneous enterotomy, cholecystectomy and fistula resection is feasible. The formation of a biliary enteric fistula can be preceded by an asymptomatic period.

## Consent

Written informed consent was obtained from the patient for publication of this case report and any accompanying images. A copy of the written consent is available for review by the Editor-in-Chief of this journal.

## Competing interests

The authors declare that they have no competing interests.

## Authors' contributions

AG conceived the case report, drafted and revised the manuscript and the relevant literature. He also was responsible for our patient's gastroenterological management. JZ was responsible for our patient's surgical management and for editing the manuscript. GW was responsible for the radiological findings and provided the CT scan figure. BH was responsible for the coordination and supervision of our patient's gastroenterological management and manuscript editing. All authors read and approved the final manuscript.
